# Perspectives on the Gamification of an Interactive Health Technology for Postoperative Rehabilitation of Pediatric Anterior Cruciate Ligament Reconstruction: User-Centered Design Approach

**DOI:** 10.2196/27195

**Published:** 2021-08-27

**Authors:** Michael McClincy, Liliana G Seabol, Michelle Riffitts, Ethan Ruh, Natalie E Novak, Rachel Wasilko, Megan E Hamm, Kevin M Bell

**Affiliations:** 1 Department of Orthopaedic Surgery University of Pittsburgh Medical Center Pittsburgh, PA United States; 2 University of Pittsburgh School of Medicine Pittsburgh, PA United States; 3 Department of Bioengineering University of Pittsburgh Pittsburgh, PA United States; 4 Department of Orthopaedic Surgery University of Pittsburgh Pittsburgh, PA United States; 5 Center for Research on Healthcare Data University of Pittsburgh Pittsburgh, PA United States

**Keywords:** IHT, pediatric, sports medicine, ACL, orthopaedics, rehabilitation, health technology, gamification

## Abstract

**Background:**

Pediatric and adolescent athletes are a large demographic undergoing anterior cruciate ligament reconstruction (ACL-R). Postoperative rehabilitation is critical, requiring patients to complete home exercise programs (HEPs). To address obstacles to HEP adherence, we developed an interactive health technology, *interACTION (iA)*, to monitor knee-specific rehabilitation. *iA* is a web-based platform that incorporates wearable motion sensors and a mobile app that provides feedback and allows remote monitoring. The Wheel of Sukr is a gamification mechanism that includes numerous behavioral elements.

**Objective:**

This study aims to use a user-centered design process to incorporate behavioral change strategies derived from self-management theory into *iA* using the Wheel of Sukr, with the aim of influencing patient behavior.

**Methods:**

In total, 10 athletes aged 10-18 years with a history of ACL-R were included in this study. Patients were between 4 weeks and 1 year post–ACL-R. Participants underwent a 60-minute triphasic interview. Phase 1 focused on elements of gaming that led to high participation and information regarding surgery and recovery. In phase 2, participants were asked to *think aloud* and rank cards representing the components of the Wheel of Sukr in order of interest. In phase 3, the patients reviewed the current version of *iA*. Interviews were recorded, transcribed, and checked for accuracy. Qualitative content analysis segmented the data and tagged meaningful codes until descriptive redundancy was achieved; next, 2 coders independently coded the data set. These elements were categorized according to the Wheel of Sukr framework. The mean age of participants was 12.8 (SD 1.32) years, and 70% (7/10) were female. Most participants (7/10, 70%) reported attending sessions twice weekly. All patients were prescribed home exercises. Self-reported HEP compliance was 75%-100% in 40% (4/10), 50%-75% in 40% (4/10), and 25%-50% of prescribed exercises in 20% (2/10) of the participants.

**Results:**

The participants responded positively to an app that could track home exercises. Desirable features included exercise demonstrations, motivational components, and convenience. The participants listed sports specificity, competition, notifications, reminders, rewards, and social aspects of gameplay as features to incorporate. In the Wheel of Sukr card sort exercise, motivation was ranked first; self-management, second; and growth, esteem, and fun tied for the third position. The recommended gameplay components closely followed the themes from the Wheel of Sukr card sort activity.

**Conclusions:**

The participants believe *iA* is a helpful addition to recovery and want the app to include exercise movement tracking and encouragement. Despite the small number of participants, thematic saturation was reached, suggesting the sample was sufficient to obtain a representative range of perspectives. Future work will implement motivation; self-management; and growth, confidence, and fun in the *iA* user experience. Young athlete ACL-R patients will complete typical clinical scenarios using increasingly developed prototypes of the gamified *iA* in a controlled setting.

## Introduction

### Background

Anterior cruciate ligament (ACL) injury can lead to pain, knee instability, and variable disability level, from reduced participation in sports to more severe limitations in activities of daily living [1,2]. ACL reconstruction (ACL-R) is the sixth most common orthopedic surgical procedure in the United States [3]. The goals for ACL-R include restored knee stability and return to prior activities. Recent studies evaluating insurance databases have noted that pediatric and adolescent athletes (young athletes) are the largest demography of patients per capita undergoing ACL-R [4,5]. Young athletes also demonstrate higher reinjury rates following ACL-R, partly because of their high level of functional demands following surgery. Biomechanical studies note that persistent muscular weakness or dysfunction is a significant risk factor for reinjury after the patient has returned to preprocedure activities and that postoperative rehabilitation is critical in reducing these negative outcomes [6,7].

Rehabilitation—specifically restoration of motion, optimizing muscular strength, and improving neuromuscular control—is demonstrably important in optimizing the quality of life of young athletes and function following ACL-R [6-8]. Adequate rehabilitation requires patients to complete prescribed exercises daily, primarily in the form of home exercise programs (HEPs) [9]. Unfortunately, due to lack of education, communication, and oversight, many patients do not fully understand or appreciate the importance of their HEP [10]. Particularly, in a young athlete population, low rehabilitation adherence is a common and critical obstacle in ACL-R recovery [8,11]. Inadequate rehabilitation can result in poorer clinical outcomes, including reduced quality of life, increased pain, and increased chances of reinjury [12-14]. Commonly reported barriers to rehabilitation adherence in the ACL-R population include fear of pain or reinjury, low self-motivation (self-efficacy) or exercise level, and a feeling of low patient control have been cited as common barriers faced by patients following ACL-R [13].

**Figure 1:**
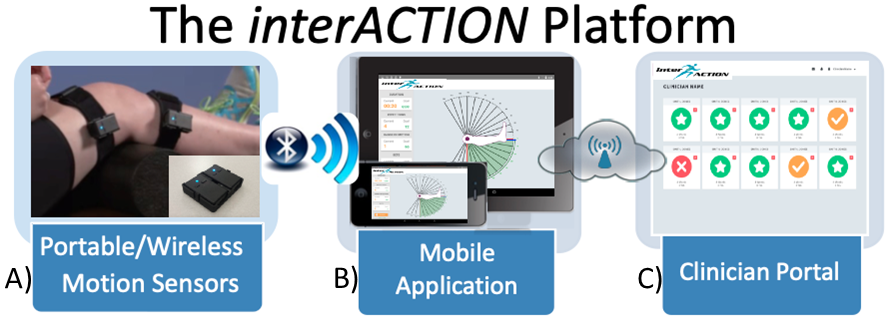
*interACTION* (*Elizur*) platform components: (A) wireless motion sensors attached to the knee of a patient; (B) screenshots from the current version of the mobile app; (C) screenshot from the current version of the clinician portal. iA provides real-time exercise feedback to the patient and logs performance metrics for remote clinicians to allow home exercise program monitoring.

In response to these obstacles, our group developed an interactive health technology (IHT) called *interACTION* (*iA; Elizur*), to prescribe and monitor home exercises for knee-specific rehabilitation. *iA* is a web-based rehabilitation platform that incorporates wearable motion sensors with a mobile device app, using real-time motion tracking to provide exercise biofeedback to the patient and log HEP performance metrics for remote clinicians (Figure 1). Through these mechanisms, *iA* helps to bridge the gaps in current physical therapy practices by allowing remote monitoring of HEP compliance and performance. *iA* has been evaluated in both healthy controls and adult patients with total knee replacement, which established a proof of concept of the technology [15].

**Figure 2:**
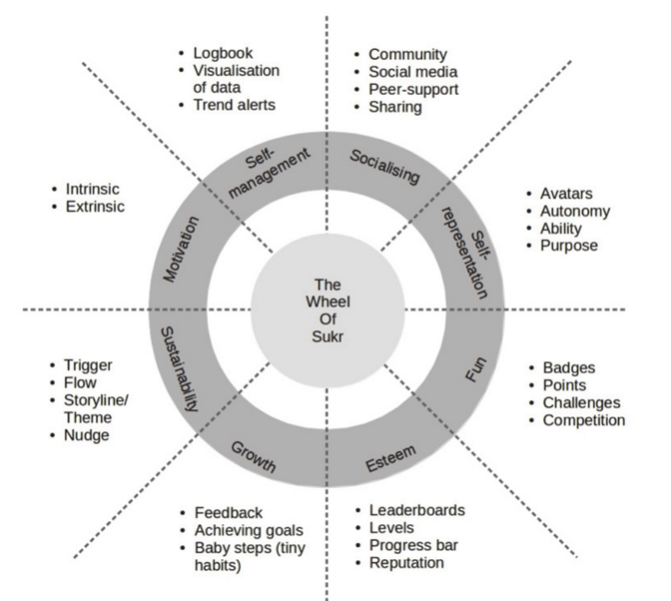
The Wheel of Sukr—a conceptual scaffold for integrating behavioral change and gamification.

Similar to many IHTs, *iA* in its current form is not grounded in models of behavioral change that may translate to a prolonged influence on patient behavior. IHTs founded on theoretical models of behavioral intervention, supported by technology, have been shown to promote exercise adherence more effectively [16-18]. The Wheel of Sukr is a proposed mechanism to introduce behavioral intervention concepts into IHTs. The Wheel of Sukr (Figure 2), which was initially described in its application to diabetes self-management, provides a structure for the incorporation of numerous behavioral elements (ie, motivation, self-management, fun, and sustainability) into a game-like experience of an IHT [19]. This process of IHT *gamification* introduces behavioral intervention concepts through techniques typically used in game development [20]. It seeks to insert concepts such as engagement, reward, and incentive into the IHT platform to better engage patients and affect behavioral change [21]. Adolescent athletes typically note prolonged (6-12 months) recovery following ACL-R as a hurdle to rehabilitation adherence, so the focus of Wheel of Sukr on sustainability was felt to be of relevance to this patient cohort [11].

### Objective

The objective of this study is to use a user-centered design process to incorporate behavioral change strategies derived from self-management theory into the *iA* platform using the Wheel of Sukr gamification framework [19]. With the implementation of gamification on the *iA* platform, we hope to better engage young athlete ACL-R patients to improve their adherence to postoperative rehabilitation programs and subsequently improve their recovery. Self-reported questionnaires focus on patient adherence and barriers to adherence are also recorded to help characterize this patient population and to inform the development of behavioral change strategies.

## Methods

### Patient Recruitment

Young athletes aged 10-18 years with a history of ACL injury and surgical reconstruction were included in this study. Patients were identified for study inclusion by reviewing the electronic medical records at our institution. All patients were between 4 weeks and 1 year from the ACL-R before their consideration for study participation. Concomitant procedures, such as meniscal repairs and chondroplasties, performed at the time of ACL-R did not exclude participation but patients undergoing multiple-ligament reconstructions were excluded. Eligible patients were contacted by a research coordinator, at which time a thorough description of the study was presented, and informed consent was obtained from the patient or guardian.

### Patient Information

After the participants were enrolled and informed consent was obtained, their age, sex, site of surgery (right or left), surgical procedures performed (including concomitant procedures), and date of surgery were recorded from the electronic medical record. On their interview date, participants were presented with a set of self-reported questionnaires, which were completed by either the participant or their proxy (parent or legal guardian). The questionnaire included demographic questions as well as the Exercise Adherence Rating Scale 10 items (EARS-10), General Self-Efficacy (GSE) scale, Tampa Scale for Kinesiophobia (TSK), and Numeric Pain Rating Scale (NPRS). The EARS-10 assesses adherence to prescribed HEPs by asking participants to answer questions about their exercise adherence and attitudes toward exercise.

GSE measures a participant’s perceived self-efficacy. Scores predict the self-perceptions of self-efficacy. The participants rated their perceived self-efficacy, and higher self-efficacy was correlated with emotion, optimism, and better health outcomes. The TSK assesses fear of movement, fear of physical activity, and fear avoidance. A score <37 is classified as *low fear*, and a score >37 is classified as *high fear* [22].

Descriptive statistics were used to summarize patient demographics and the results for the EARS-10 GSE, TSK, and NPRS. The Pearson correlation coefficient (*r*) was calculated between these patient-reported outcome measures and was interpreted as follows: 0-0.1, negligible; 0.1-0.3, weak; 0.3-0.7, moderate; 0.7-1.0, strong .

### Interview Process

The patient interviews were performed in private rooms in a clinical research suite, which served as a controlled environment to simulate outpatient medical encounters. The participants took part in a single individual interview with three distinct phases, as described below. The audio of participants was recorded throughout the entirety of their interviews.

Phase 1 followed a standardized, semistructured script, with open-ended questions designed to facilitate conversation with a specific focus on: (1) identifying critical elements of existing gaming experiences (ie, sports, video games) that lead to high levels of participation (adherence) and (2) eliciting experiential information regarding their knee surgery and subsequent physical therapy.

In phase 2, participants were presented with a series of cards, each presenting an aspect of game play designed to promote continued interest and participation. Each card displayed an individual component and a description of the Wheel of Sukr methodology. The language was altered to the appropriate reading level of the participants. The participants were prompted to *think aloud* as they reviewed these cards and were prompted to rank the cards in order of importance in maintaining interest in a game. The final card rankings of the participants were recorded. The card sort was followed by a brief semistructured survey to elucidate the motivations behind the card sort results.

Finally, for phase 3, participants were presented with the current version of the *iA*. Participants were again prompted to *think aloud* as they had experienced the device. The participants were then presented with a brief semistructured survey to determine their overall opinion of the current device, elicit suggestions for improvement, and identify any gamification changes that should be implemented.

Phase 1 of the interviews was performed by an experienced facilitator with extensive background in qualitative research methods from the Qualitative Research Core at the University of Pittsburgh Center for Research on Healthcare Data. Phases 2 and 3 of the interviews were performed by a study research assistant, as they did not require the same level of experience with qualitative interview facilitation as the first phase. The interviews were audio recorded, transcribed, checked for accuracy, and transferred to ATLAS.ti (ATLAS.ti Scientific Software Development, GmBH) for coding.

### Qualitative Data Analysis

A qualitative codebook was developed by the phase 1 interviewer using an inductive approach and shared with the study team to ensure that it captured key concepts that were of interest to the investigators. Once the codebook was finalized, 2 Qualitative Research Core coders used ATLAS.ti to independently code the full data set and to reconcile any coding differences. The interviewer or primary coder then conducted a content analysis using the coded data [23]. The resulting content analysis was confirmed by the secondary coder in the form of investigator triangulation.

### Additional Data Analysis

The research team assisted in developing the best ways to display, represent, interpret, triangulate, and summarize the qualitative findings to achieve a fully integrated description [24]. These elements were categorized according to the Wheel of Sukr framework and translated into elements relevant to improving the design of *iA* to increase ACL-R patient engagement.

## Results

### Overview

A total of 10 interviews were conducted during the fall of 2019. Participants ranged in age from 11 to 15 years (mean 12.8, SD 1.32 years). Participant demographics are presented in Table 1. All 10 participants completed all three phases of the interviews.

Results of the EARS-10, GSE, and TSK questionnaires are presented in Table 2. The median value for the EARS-10 questionnaire was 26, with values ranging from 6 to 36. The median self-efficacy rating was 31.5, participants reported values ranging from 21 to 40, with 40 being the maximum value on the scale. Participants were divided evenly between low and high fear on the kinesiophobia scale. The median rating was 37.5 with ratings ranging from 19 to 44.

**Table 1:** Participant demographic information (N=10).

Demographics			Values
**Gender, n (%)**			
	Male	3 (30)	
	Female	7 (70)	
Age (years), mean (SD)			12.8 (1.32)
**ACL^a^ graft, n (%)**			
	Quadriceps tendon	1 (10)	
	Iliotibial band	1 (10)	
**Other procedures, n (%)**			
	Left knee arthroscopy	1 (10)	
	Lateral meniscal repair	1 (10)	
	Medial and lateral meniscal repair	1 (10)	
**Surgery date, n (%)**			
	February-April 2019	2 (20)	
	May-July 2019	5 (50)	
	August-October 2019	3 (30)	
**Time since surgery (months), n (%)**			
	0-2	2 (20)	
	2-4	3 (30)	
	4-6	3 (30)	
	>6	2 (20)	
**PT^b^ start date, n (%)**			
	March-May 2019	2 (20)	
	June-August 2019	5 (50)	
	September-November 2019	2 (20)	
	Not recorded	1 (10)	
**PT duration, n (%)**			
	3 months	1 (10)	
	Ongoing	9 (90)	
**PT frequency, n (%)**			
	Once every 2 weeks	1 (10)	
	Once a week	1 (10)	
	Twice a week	6 (60)	
	Once or twice a week	1 (10)	
	Thrice a week	1 (10)	
**HEP^c^ format, n (%)**			
	Verbal	7 (70)	
	Hard copy	7 (70)	
	Mobile app	2 (20)	
**HEP completed (%), n (%)**			
	25-50	2 (20)	
	50-75	4 (40)	
	75-100	4 (40)	

^a^ACL: anterior cruciate ligament.

^b^PT: physical therapy.

^c^HEP: home exercise program.

**Table 2:** Patient-reported outcomes of Exercise Adherence Rating Score, General Self-Efficacy score, and Tampa Scale of Kinesiophobia (TSK) listed by participant. Participants divided into low fear and high fear categories based on results of TSK tabulation.

Participant	EARS-10^a^ score	GSE^b^ scores	TSK^c^ score
1	25	36	*19* ^d^
2	21	31	39
3	21	36	*37*
4	17	24	*37*
5	34	30	38
6	27	31	44
7	36	39	*29*
8	33	40	42
9	6	21	*35*
10	36	32	42

^a^EARS-10: Exercise Adherence Rating Scale 10 item; median score 26 (range 6-36).

^b^GSE: General Self-Efficacy; median score 31.5 (range 21-40).

^c^TSK: Tampa Scale of Kinesiophobia; median score 37.5 (range 19-44).

^d^Values in italics denote low-fear patients (defined as Tampa Scale of Kinesiophobia score ≤37).

NPRS scores showed moderate rates of postoperative pain, which improved with the start of physical therapy and study enrollment (Table 3). The median pain rating postoperatively was 4, with postoperative pain ranging from 0 to 7. Pain at the start of physical therapy ranged from 0 to 6, with a median value of 2. Pain in the 24 hours before the interview was low, with a median score of 0 and a range from 0 to 2.

**Table 3:** Numeric Pain Rating Scale responses, demonstrating mild-to-moderate postoperative pain that improved during and following physical therapy (N=10).

Pain rating scale responses			Participants, n (%)
**Pain with surgery**			
	None	2 (20)	
	Mild	4 (40)	
	Moderate	4 (40)	
	Severe	0 (0)	
**Pain during physical therapy**			
	None	2 (20)	
	Mild	7 (70)	
	Moderate	1 (10)	
	Severe	0 (0)	
**Pain in the last 24 hours**			
	None	8 (80)	
	Mild	2 (20)	

Pearson correlation coefficients were calculated for patient-reported outcomes. The correlation between EARS-10 and GSE was 0.79, indicating a strong correlation. Another strong correlation was found between the NPRS surgery outcome and the TSK, with a value of 0.74. Both these correlations had a significant *P* value. All other *P* values were not significant. The *r* value for NPRS at the beginning of PT and EARS-10 was calculated as −0.46, a moderate correlation (Table 4).

**Table 4:** Pearson correlation coefficients between patient-reported outcome measures. Strength of correlation increases as the *r* value approaches 1.

		GSE^a^	TSK^b^	NPRS^c^-surgery	NPRS-physical therapy	NPRS-24 hours
**EARS-10^d^**						
	*r*	0.792	0.101	−0.257	−0.456	0.173
	*P* value	.006	.78	.47	.19	.63
**GSE**						
	*r*	—^e^	−0.207	−0.227	−0.280	0.345
	*P* value	—	.57	.53	.43	.33
**TSK**						
	*r*	—	—	0.741	0.1284	0.277
	*P* value	—	—	.01	.72	.44

^a^GSE: General Self-Efficacy Scale.

^b^TSK: Tampa Scale for Kinesiophobia.

^c^NPRS: Numeric Pain Rating Scale.

^d^EARS-10: Exercise Adherence Rating Scale 10 item.

^e^Not applicable.

### Phase 1: Semistructured Interview

#### Injury and Surgical Recovery Experience

The participants typically injured their knees while playing a sport. Basketball was the most common sport, with other injuries occurring during soccer, lacrossing, skiing, and baseball. Participants described their surgical experience as *not bad* or said that it was tough at first and got better over time. Most patients did not complain of pain. Those who mentioned pain said they used counter pain medicine when they felt pain. As one patient explained:

Um, it was difficult, but it got easier along the way...Um, I wasn’t in that much pain after surgery. Um, I took Tylenol if I had any pain.

#### Experiences in Postoperative Physical Therapy

Most participants attended all physical therapy sessions. Those who missed a session were missed because of being sick or having another appointment. One participant reported at the time of their interview that they had finished their physical therapy 1 month prior. When asked about their physical therapy experience, participants described that their therapy started out easily and progressively became more challenging:

Um well at first I just started like trying to bend my knee a little bit more and do some easier exercises and then it got like a little bit harder and...the exercises were like more difficult...at first it was super easy, and we didn’t do a lot of stuff but then we started doing more stuff and it like got more challenging.

All participants were prescribed home exercises by their physical therapists. For the most part, they completed their home exercises. Others who missed a day of home exercise said they did not feel like it, they forgot, or were busy, which was consistent with responses on the EARS-10. Most said that their physical therapist did not monitor their home exercises, instead citing their parents as monitors. A few mentioned that while at a physical therapy session, the therapist would ask about their home exercises. Most also said that it would have been beneficial if their therapist monitored their home exercises. Select responses included:

Yeah I feel like it could be more uh motivating too...Um because the patient would know that the physical therapist could tell if you were doing your exercises or not and then they obviously wouldn’t want the physical therapist to know that they’re not doing them

Uh, because I feel like if he would have monitored it, um, they would have known where I was quicker, to help reach my goals.

The most common reason that the participants wanted to continue therapy was to improve their knee function and return to activities. Select responses include:

Just to regain–to go back to sports mainly; that’s the main reason why I go, but also just to regain, like, stability in my knee.

Well, I wanna get back to sports as fast as I can and I wanna have my leg be as strong as before.

Others mentioned acceptance that they did not have a choice when it came to performing physical therapy during recovery and that the progress they were making was motivation to continue:

Yeah I-I didn’t really have a choice. [laugh]...Because like if I didn’t do it then I wouldn’t get better. And I’d rather be better than be like that for all the rest of my life.

#### Preferred Activities and Games

When prompted to provide examples of activities requiring dedicated practice in their daily lives, the majority of participants noted that they played a sport or multiple sports. Other less commonly cited activities included musical instruments or foreign languages. The reasons cited for continued practice for these activities were varied and included: the activities are fun or enjoyable or personally motivating, encouraging physical activity, and satisfying desires for both social interaction and competition. Sample responses included, “it’s just fun...being with friends and...working out,” “I like playing it...competing against other teams,” “also because of the team aspect...being a part of something,” and “I just have inner motivation to do them, like it’s something that I enjoy.”

Participants cited losing interest, lacking talent or ability, losing companions or teammates, and parental influences as reasons for withdrawal from activities. Many participants noted that they would keep doing an activity that they personally no longer care about with the help of extrinsic motivators such as friends, family members, or a coach to encourage them:

Maybe I’d do something if, like, my friends were doing it.

...help from others or maybe like...someone to, someone else to motivate...probably friends, my sister, parents, a teacher, or a coach depending on what the activity is.

A few mentioned acceptance that they just have to do an activity they do not care about (most commonly school), whereas others also said they would keep doing an activity they did not care about because it keeps them active.

All participants said that they enjoyed playing games. The types of games they enjoyed were sports, board games, and videogames. Most of the participants said that sports were their favorite games to play. The reasons they played their favorite games were enjoyment, competition, social aspects, and the nature of the game—or how the game is played, and its aspects are what they like about it. When asked how frequently they would play their favorite games, some said almost every day or every day. Typically, this frequency was for sports-related activities such as practices, games, or casual play. Some others said that a few times a week, a few participants said once a week. The frequency of how often they played their favorite games depended on whether it was a sport, board game, or videogame—with sports having more frequent sessions.

#### Mobile Apps

Participant responses were positive when asked about using a mobile app for home exercises. They thought that an instructional component on the app would be helpful: “...if I had that thing on my leg it would just make me feel like I’m actually doing something and it would make me feel like it’s easier and it would tell me if like I need to do better or not.” They also felt that the app would be motivational and help keep them accountable when it came to completing their home exercises:

that would help a lot because again the physical therapist would be able to tell. And I also feel like you would get more uh research and like knowledge out of that.

Participants also cited convenience as it would be accessible by their phone, “I think it would be really convenient especially since you have your phone on you pretty much 24/7.”

Participants were prompted to list features from their favorite games that they would include in a mobile app for their physical therapy. Sports specificity was commonly cited, such as:

Like, the soccer...foot skills...what skills I could do while I’m in physical therapy and like what could possibly help.

...it would also help motivate I feel like if you were practicing for a-I mean if you were training for a particular uh like sport or event or something like that.

Other features from the participants’ favorite games that they endorsed included competition, notifications or reminders, rewards, and social aspects of gameplay:

If it had notifications or like reminders set for your uh for-what you have to do and if it just explained it like thoroughly how to do each thing having like your goals maybe like a chart of how far you’ve gone.

Maybe if there was, like, a competitive part of it where like...you, like, have to reach this goal, then, but if, like, a little hard to reach so you have to, like, kind of push yourself out of your comfort zone to do all of the exercises.

### Phase 2: Wheel of Sukr Card Sort

In the Wheel of Sukr card sort exercise, the collective top three themes were motivation, self-management, and esteem (when using the median). When using the mean, motivation was first; self-management was second; and growth, esteem, and fun tied for the third. The top three themes of each participant are presented in Table 5, along with their reported favorite games, and the reasons and motivations for playing their favorite game. We presented the data individually by participants because the participants generally did not describe the cards any further than what it already said on the cards themselves—that is, most did not provide information on why they liked a concept beyond how it was already described on the card, and thus it was not possible to derive themes from their descriptions.

**Table 5:** Wheel of Sukr themes and corresponding motivation among participants. Chosen themes, favorite games, and underlying motivations listed by participant.

Participant	Wheel of Sukr themes	Favorite games	Reasons and motivations
1	Motivation Fun Esteem	Baseball Football Monopoly	Fun and physical activity
2	Growth Fun Self-management	FIFA^a^ Fortnight Minecraft Monopoly	Like soccer, multiple modes of play
3	Motivation Fun Growth	Strategy games Card games Sports	Focus and thinking, being with other people
4	Sustainability Motivation Socializing	Videogames	Calming and fun
5	Socializing Self-representation Self-management	Board games	Fun to play with friends and family
6	Motivation Esteem Self-management	Team sports	Support from team and being active
7	Fun Motivation Self-management	Basketball Board games	Playing with friends, competition
8	Motivation Esteem Self-management	Monopoly	Competitive
9	Self-management Growth Esteem	Sports or hockey	Fun to play, interesting, fast paced
10	Growth Self-representation Motivation	Soccer	Enjoy it

^a^FIFA: Fédération Internationale de Football Association.

When talking about what elements or features from the cards the participants would like to see in a game, many referenced their top three. Few participants mentioned a feature of a card, not in the top three. Textbox 1 lists the example recommendations for each card theme provided by the participants.

**Example recommendations for incorporation into future game or app matched to respective Wheel of Sukr card elements.**

**Card Element and Example Recommendation**

- Motivation
  Position on a leaderboard
  Reward for playing each day
  Points for completing tasks
  Leveling up
- Self-management
  Seeing daily results
  Setting goals
- Socializing
  Connecting to friends in the game
- Self-representation
  Creating own character
- Fun
  Challenges within the game
- Esteem
  Congratulations
- Growth
  Complete goals
  Increased challenge progression
- Sustainability

### Phase 3: iA Demonstration

#### Review of Current Version

Participants generally had a favorable review of the current version of *the iA*. Participants liked that the device would be able to show them how they are moving during their physical therapy exercises:

I like the, how it shows you how far you have to go and stuff with like the angles and stuff. It’s, it looks pretty simple and I like how it has the videos and it tells you everything.

Other participants elaborated that it would help them because they would not have to wait until their next physical therapy session to get feedback about their exercises:

That’s so helpful...at the beginning [my therapist] tried to set goals but I couldn’t really tell if I was achieving, like, the certain angle and then...I’m type who worries about everything, so I thought that I would, like, overstretch it or something like that. So I would one hundred percent use

iA

Overall, all participants liked the device or thought it was *cool*. All would like to have something like it at home for physical therapy exercises. Some participants noted that they appreciated having the ability of a physical therapist to monitor their progress remotely:

I think that would be helpful. And then, maybe, like...they could send you feedback once a week or something before your next session.

Whenever a parent spoke during the demo, they commented positively about the device and app. One parent said that it would help with motivation: “I think it would definitely help motivate, you know, an athlete to, to continue to do it because, you know, a lot of ‘em get depressed and stuff and don’t wanna do anything.” Most participants did not have negative criticisms about the device or app, but a few felt that the sensor straps were cumbersome or irritating, suggesting a sleeve or other method of fixation.

#### Suggestions for Future Improvement

The participants verbalized various suggestions or improvements for the device and/or app related to understanding the exercises, creating goal-motivated incentives to complete exercises, and visual additions or changes. From a usability standpoint, some suggested improved descriptions of the individual exercises, whereas others focused on improving the method of attaching the sensors to their knees. One individual suggested the inclusion of a general surgical recovery guideline:

[some]thing where you could search up if you had a question about [your recovery]...just like if you were feeling something it’d be cool to see if it was normal. Because, like, sometimes I felt, like, a pop or something, and...I would have to wait until the next time to ask [my therapist]. But if there’s, like, a way to search up something that you’re feeling.

The features of gameplay that participants wished to incorporate closely followed the themes from the Wheel of Sukr card sort activity. Suggestions for improvements centered more on mobile apps and games as opposed to sports and may have been influenced by phase 2’s focus on these topics. Some participants suggested the incorporation of a sport into the *iA* app, but none went further to elaborate specific ways to incorporate sports. Participants also did not specifically cite social networking capabilities as essential additions to *iA*, but they noted this capability to be of interest or importance in general mobile apps or games. Textbox 2 shows the suggested *iA* improvements separated into their respective Wheel of Sukr categories.

**Specific suggested iA improvements organized by Wheel of Sukr categories.**

**Wheel of Sukr Category (Suggested Improvement)**

- Motivation
  Encouragement
  Tips
  Reward or congratulations for completion
- Self-management
  Visual progress graph or timeline
- Socializing
- Self-representation
  Create-a-character option with customizable extras
- Fun
  Earning points or prizes for completion
  Selection of games
  Bonus points and incentives for completing exercises or improvements
- Esteem
- Growth
  Timeline demonstrating baseline to current progress
- Sustainability

## Discussion

### Principal Findings

This study is the first in a stepwise, user-centered design process to integrate behavioral theory concepts into the design of *iA*, a mobile IHT to prescribe and monitor home exercises for knee-specific rehabilitation following ACL-R in young athletes. Participants reported that their favorite games were sports, with many participants reporting playing multiple sports and most participants becoming injured through their sports. Participants generally reported positive experiences with PT following ACL-R, yet most thought *iA* and its remote monitoring would have been a helpful addition to their overall PT experience and recovery.

When considering gameplay and mobile apps, the Wheel of Sukr domains of motivation, self-management, and confidence were deemed of highest importance, by median. The top three domains were motivation, self-management, and growth, esteem, and fun tied for third. Participants’ impressions of a device that tracked their knee PT exercises were overwhelmingly positive. The participants want the app to include a feature that tracks their exercise movement for accuracy and to include features to encourage or motivate them.

On the basis of these interviews, future versions of *iA* will strive to incorporate the following components of gameplay:

1. Provide a connection to favorite games or activities, which are predominantly sports in this young athlete population.
2. Provide goal- and incentive-based biofeedback during rehabilitative workouts to provide a source of motivation, both extrinsic and intrinsic, to complete assigned tasks and expedite recovery.
3. Provide a reward program based on progression along an expected rehabilitation trajectory with thresholds for performance and ability to socialize with fellow users (avatars, leaderboards, etc).
4. Provide motivational and/or encouragement components (alerts, clinician feedback, background educational information, etc) that draw users into the app daily.

Having a connection to favorite games or sports would help establish interest in the app from a preexisting interest. Incorporation of sport-specific activities later in the rehabilitation process may be helpful to promote longitudinal usage of the app:

...it would obviously help the knee get better for that specific sport so you’d be ready for that sport...And it would also help motivate I feel like if you were practicing for...a particular...sport or event.

The participants wanted to know that they were doing their home exercises correctly. This feature is already present in *iA*, through real-time biofeedback provided by the wireless sensors, but in its current form, it acts as a simple counting mechanism with no gamified structure. By improving this interface, the app can show how their knees are moving and provide more motivational feedback during workouts. The participants also wanted a better sense of how their recovery progressed. Incorporating performance thresholds or milestones and rewards, allowing users to interact with peers via avatars, and integrating regular feedback from their remote clinicians will be explored in future studies. This would improve self-management capabilities and provide extrinsic sources of motivation.

A strong significant correlation was calculated between the EARS-10 survey measuring exercise adherence and the GSE survey measuring self-efficacy. This strong correlation indicates that individuals with low self-efficacy are more likely to have lower adherence to HEPs. As the average score of the GSE was 31, with a value of 40 being the maximum score and indicating the highest level of self-efficacy, the participants interviewed overall had a fairly high self-efficacy score. Confidence and self-management were both highly rated components of the Wheel of Sukr and ideas related to a high level of self-efficacy. Designing the next iteration of *iA* and taking into account the highly ranked ideals of confidence and self-management can keep those with high self-efficacy adherent to their maximum potential and engage young athletes with naturally lower self-efficacy.

A strong significant correlation between a high NPRS value postoperatively and a high rating on the TSK was calculated, suggesting that higher pain postsurgery is related to a fear of movement. This finding is consistent with those of previous studies on chronic musculoskeletal pain and kinesiophobia using the Tampa Scale [25]. Goal and incentive-based feedback, both intrinsic and extrinsic, will be implemented in future iterations of *iA* to keep users engaged in the early aspects of their rehabilitation despite high levels of pain. Starting the rehabilitation process out quickly postoperatively and having patients adhere to their HEP over time should decrease pain levels and kinesiophobia as the patient progresses through treatment, as patients will gain more confidence as exercises and movement become easier and less painful. In addition, direct communication from remote clinician monitoring progress can ease patient hesitation and build confidence by answering questions and providing encouragement during the rehabilitation process.

A moderate negative correlation was found between the EARS-10 and NPRS-PT ratings, indicating that higher pain during physical therapy is related to lower home exercise adherence. This correlation was not statistically significant; therefore, future work is needed to completely understand this relationship. The patients do not want to perform their assigned exercises on their own if they are in pain. Increasing patient adherence through motivational tools to draw users to the app daily will increase the frequency of home exercise performance and decrease pain over time as the knee begins to return to its previous level of function.

*iA* is an IHT designed to bridge the gap between patients and clinicians during rehabilitation for knee injuries and surgeries. The use of IHTs to promote health and manage illness has led to fundamental changes in health practices and transformed health care [26]. Qualitative studies indicate that patients have positive perceptions of IHTs and their benefits, including awareness of progress, feeling of being in control, empowered self-management, and improved patient-clinician communication [27-30]. IHTs founded on theoretical models of behavioral intervention supported by technology have been shown to promote exercise adherence more effectively than those devoid of behavioral modeling [16-18]. The Wheel of Sukr method was applied to *iA* as it is designed to affect behavioral changes for chronic conditions, and many adolescent athletes view their ACL-R rehabilitation as a long-term event and cite fatigue as a prime driver of noncompliance [11,21,31].

### Limitations and Future Considerations

This study has several limitations. Given that qualitative studies emphasize exploration of participant perspectives in great depth, sample sizes tend to be small, potentially limiting generalizability. In addition, there may be bias in the sample, insofar as participants may have agreed to be interviewed because of a particular interest in the topic. However, thematic saturation was reached by the conclusion of our 10 interviews, suggesting that the sample was sufficient to obtain a representative range of perspectives and experiences. The interview responses were monitored by a quantitative data expert who determined when thematic saturation was achieved. Furthermore, our sample was diverse in terms of gender, age, and surgical procedures (concomitant procedures with ACL-R).

Gamification of *iA* was explored in this study to incorporate the behavioral theory concepts of gaming to improve engagement with and adherence to the *iA* rehabilitation platform, which can be applicable to other IHTs as well [19,21]. Moving forward, we will focus on improving the domains of motivation, self-management, and growth or esteem or fun to the *iA* user experience. In subsequent studies, young athlete ACL-R patients will be asked to complete typical clinical scenarios using progressively more developed prototypes of the gamified *iA* in a controlled laboratory setting. The usability of each gamification aspect and its ability to induce continued user engagement will be assessed objectively and subjectively. On the basis of these metrics, *iA* will undergo an iterative improvement process to address its current limitations.

## Abbreviations

ACL: anterior cruciate ligament
ACL-R: anterior cruciate ligament–reconstruction
EARS-10: Exercise Adherence Rating Scale 10 item
GSE: General Self-Efficacy
HEP: home exercise program
iA: interACTION
IHT: interactive health technology
NPRS: Numeric Pain Rating Scale
TSK: Tampa Scale for Kinesiophobia
